# Health-related quality of life before and during the COVID-19 pandemic in Switzerland: a cross-sectional study

**DOI:** 10.1007/s11136-023-03414-0

**Published:** 2023-04-21

**Authors:** Katharina Roser, Julia Baenziger, Anica Ilic, Vera R. Mitter, Luzius Mader, Daniela Dyntar, Gisela Michel, Grit Sommer

**Affiliations:** 1 Faculty of Health Sciences and Medicine, University of Lucerne, Lucerne, Switzerland; 2 Heart Centre for Children, The Sydney Children’s Hospitals Network, Sydney, NSW Australia; 3 Center for Heart Disease and Mental Health, Heart Institute and the Division of Behavioral Medicine and Clinical Psychology, Cincinnati Children’s Hospital Medical Center and the Department of Pediatrics, University of Cincinnati College of Medicine, Cincinnati, OH USA; 4 Department of Gynaecology, Bern University Hospital, University of Bern, Bern, Switzerland; 5 Institute of Social and Preventive Medicine, University of Bern, Bern, Switzerland; 6 Cancer Registry Bern-Solothurn, University of Bern, Murtenstrasse 31, 3008 Bern, Switzerland; 7 Swiss Childhood Cancer Registry, University of Bern, Bern, Switzerland; 8 Department for BioMedical Research, University of Bern, Bern, Switzerland; 9 Department of Pediatrics, Inselspital, Pediatric Endocrinology, Diabetology and Metabolism, Bern University Hospital, University of Bern, Bern, Switzerland

**Keywords:** Health-related quality of life, Mental health, Physical health, SF-36, Coronavirus, SARS-CoV-2

## Abstract

**Introduction:**

The COVID-19 pandemic forced people to give up their daily routines and adjust to new circumstances. This might have affected health-related quality of life (HRQOL). We aimed to compare HRQOL during the first COVID-19 wave in 2020 to HRQOL before the pandemic and to identify determinants of HRQOL during the pandemic in Switzerland.

**Methods:**

We conducted a cross-sectional online survey during the pandemic (between May and July 2020; CoWELL sample; convenience sample). Before the pandemic (2015–2016), we had conducted a cross-sectional paper-based survey among a representative random sample of the Swiss general population (SGP sample). In both samples, we assessed physical and mental HRQOL (Short Form-36) and socio-demographic characteristics. In the CoWELL sample, we additionally assessed health- and COVID-19-related characteristics. Data were analysed using linear regressions.

**Results:**

The CoWELL sample included 1581 participants (76% women; mean age = 43 years, SD = 14 years) and the SGP sample 1209 participants (58% women, mean age = 49 years, SD = 15 years). Adjusted for sex, age, and education, the CoWELL sample reported higher physical HRQOL (PCS, +5.8 (95% CI: 5.1, 6.6), *p* < 0.001) and lower mental HRQOL (MCS, −6.9 (−7.8, −6.0), *p* < 0.001) than the SGP sample. In the CoWELL sample, especially persons with lower health literacy, who had no support network or who have had COVID-19, reported lower HRQOL.

**Discussion:**

Aspects unique to the COVID-19 pandemic affected HRQOL. Vulnerable persons such as those having had COVID-19, less support opportunities, and with lower health literacy are especially prone to impaired HRQOL during the COVID-19 pandemic.

**Supplementary Information:**

The online version contains supplementary material available at 10.1007/s11136-023-03414-0.

## Introduction

The beginning of the coronavirus disease 2019 (COVID-19) pandemic was an exceptional situation, which was likely to affect health-related quality of life (HRQOL). Since its discovery in late 2019, the Severe Acute Respiratory Syndrome Coronavirus 2 (SARS-CoV-2) causing COVID-19 spread rapidly throughout the world with an estimated mortality rate of 1–4% [1]. By 1 January 2022, the WHO reported > 281.8 million COVID-19 cases and > 5.4 million deaths attributed to COVID-19 worldwide [2]. Authorities of most countries implemented public health protective measures to prevent spread of the virus and exhaustion of hospital capacities. In Switzerland, these measures included travel restrictions, gathering bans, and closures of childcare, schools, stores, personal services, restaurants, sport and recreational establishments, and public institutions [3, 4]. Working from home was strongly encouraged. Most people had to give up their daily routines and adjust to new circumstances. At that time, knowledge about COVID-19, its potential consequences, and its treatment options were limited. Daily life adaptions and the potential danger from COVID-19 to the personal health as well as the health of loved ones may have compromised well-being in the population.

The concept of HRQOL includes physical and mental aspects of an individual’s perceived health [5–7]. Numerous studies investigated quality of life during the pandemic, often in subpopulations, such as patients with chronic conditions [8–10], persons after a SARS-CoV-2 infection [11–14], persons under quarantine [15, 16], elderly people [17, 18], or children and adolescents [19–23]. These studies yielded differing results due to different study populations, and often lacked appropriate comparison groups, ideally using data collected before the pandemic [15, 24–32]. Moreover, most studies comparing HRQOL before and during the pandemic were conducted in subpopulations such as older adults [33], desk workers [34], or cancer patients [35]. Only few studies have included general population samples and compared HRQOL during the pandemic with HRQOL before the pandemics. In Austria, mental HRQOL was decreased after four weeks of lockdown compared to before the pandemic [36], and in US young adults, depressive symptoms were increased during compared to before the pandemic [37]. In a longitudinal study among the Japanese general population, physical and mental HRQOL decreased under pandemic conditions [38]. Because pandemic measures differed considerably between countries [39], it is important to address the impact of the pandemic on HRQOL in the general population of other countries. The current study aimed at: (1) comparing HRQOL during the first COVID-19 wave of spring 2020 to HRQOL before the pandemic in a convenience sample of the Swiss population and (2) identifying determinants of HRQOL during the pandemic.

## Methods

### Samples and procedures

#### CoWELL sample

This study is part of the larger CoWELL project assessing the socio-economic situation, psychological distress, and HRQOL of people living in Switzerland during the early phase of the COVID-19 pandemic. Using the online survey tool Qualtrics™, we distributed the survey among our personal and professional networks and on social media (LinkedIn, ResearchGate, Twitter, WhatsApp). Sampling started on 4 May 2020, and continued through 6 July 2020. Data were collected anonymously. Participants were eligible if they were at least 18 years old, living in Switzerland at time of the survey, and submitted the completed questionnaire.

#### Swiss general population (SGP) sample

We conducted a population-based survey using a random representative sample of the Swiss general population (regarding age, gender, and language region [German/French/Italian]). It consisted of individuals aged 18–75 years in 2015 and was sampled by the Swiss Federal Statistical Office (SFSO). Household members were invited individually. Study information was sent two weeks prior to sending a paper-based questionnaire, and one reminder was sent to non-respondents after four weeks.

Both surveys were available in German, French, and Italian to cover the three main language regions of Switzerland.

### Measurements

#### Health-related quality of life (HRQOL)

HRQOL was assessed by the Short Form-36 (SF-36) version 2 questionnaire. The 36 items cover the eight subscales physical functioning (PF, 10 items), physical role functioning (RP, 4 items), bodily pain (BP, 2 items), general health perceptions (GH, 5 items), vitality, (VT, 4 items), social role functioning (SF, 2 items), emotional role functioning (RE, 3 items), and mental health (MH, 5 items) [40]. As described in the manuals, we did not use the item that indicates the perceived change in health [41, 42]. The eight subscales can be further aggregated into two summary scores describing physical and mental HRQOL: Physical Component Summary (PCS) and Mental Component Summary (MCS) [40–42]. Higher scores within the subscale or the summary score indicate better HRQOL in the respective subscale or summary score. We used validated versions of the SF-36 [41] in German, French, and Italian. Translations of the SF-36 are culturally appropriate and comparable [43, 44]. The SF-36 can be completed in 5 to 10 min, has a high acceptability, and good psychometric properties in general populations and populations with chronic diseases [43–45]. We converted raw scores of the CoWELL sample into T-scores using norm-based scoring according to normative data from the Swiss general population [41, 46]. We considered meaningful differences proposed by the developer of the SF-36 version 2 with the following values for the eight scales and the two summary scores when T-scores were between 30 and 40: PF, 3; RP, 3; BP, 3; GH, 2; VT, 2; SF, 3; RE, 4; MH, 3; PCS, 2; MCS, 3 [41]. For higher T-scores, these values tend to be higher but the developer does not provide exact values.

#### Socio-demographic characteristics

In both the CoWELL and the SGP sample, we assessed the following potential socio-demographic and socio-economic determinants of HRQOL: gender (male, female, other; other was recoded to either male (*n* = 1) or female (*n* = 4) based on height using data on the average height of the Swiss population as a reference [47]), age (in years; 18–25, 26–35, 36–45, 46–55, 56–65, ≥ 66; and continuous), education (compulsory schooling or vocational training, upper secondary education, university education), language of questionnaire (German, French, Italian), employment status (employed, in education, other [persons who were retired, managing a household, seeking a job, receiving disability insurance, or other forms of occupation]), and having children aged 0–14 years in the household (no, yes). In the CoWELL sample, we categorized job type based on the General Classification of Economic Activities (NOGA) [48] (health services, essential services [jobs in agriculture, manufacturing, waste management, construction; trade, transportation, gastronomy; education; social work], office jobs [jobs in information and communication, finances, insurances, real estate; scientific and technical activities; administration; arts and other service activities], other [persons who were retired, unemployed or not actively working]), and we assessed living situation (alone, with partner, with partner and children, with parents and/or children, other situation [living in a shared apartment or other living arrangements]).

#### Health- and COVID-19-related characteristics

In the CoWELL sample only, we assessed health- and COVID-19-related characteristics using self-developed questions. We assessed: time since start of pandemic measures (16 March 2020; continuous, in days), physical distancing (isolation/self-isolation/preventive self-isolation, physical distancing, initial physical distancing/no physical distancing), contact to person who tested positive for COVID-19 (yes [confirmed or likely], no), own perception about having already had COVID-19 (yes, no), having a person to ask for support (yes, no, no need for support), contact with family and friends (not enough contact, enough contact/no need for contact), frequency of information seeking (daily, several times per week, once per week or less), risk to develop severe COVID-19 (yes, no), and health literacy (continuous score). We assessed health literacy using the validated 12-item short version (HLS-Q12) [49] of the European Health Literacy Survey Questionnaire (HLS-EU-Q) [50]. The HLS-Q12 measures health literacy across three health domains and four cognitive domains [49]. We added one additional item to the HLS-EU-Q (item 12) because we considered it important for health literacy associated with COVID-19 (“How easy would you say it is to judge if the information about illness in the media is reliable?”).We defined a binary variable for the risk of developing severe COVID-19 (yes, no) based on established risk factors according to the Swiss National Science Task Force [51]. Participants with a BMI > 30 kg/m^2^ (calculated as weight in kilograms divided by height in metres squared) or who reported to have a pre-existing chronic condition (including cardiovascular diseases, lung diseases, diabetes, hypertension, history of cancer) or a transplant were considered at risk of developing severe COVID-19.

Data included in this study were from questionnaires filled in between 6 May 2020 and 29 June 2020. At the time of data collection, first relaxations of protective measures were introduced, e.g. openings of obligatory schools, restaurants, sport facilities, museums (11 May 2020), permission of small meetings, events and demonstrations, in-classroom teaching in educational facilities, openings of touristic facilities, further lifting of travel restrictions to most neighbouring countries (6 June 2020), and permissions of gatherings with unlimited number of participants (22 June 2020). The Federal Office of Public Health published all details on measurements and their relaxations on their website [52].

### Statistical analysis

#### Handling of missing data

We substituted missing SF-36 data with the average score of completed items in a subscale, if the participant answered at least 50% of items in the respective subscale, as recommended by the developer of the SF-36 [45]. Ninety per cent of observations had complete information in all socio-demographic and health- and COVID-19-related variables. Job type had the highest percentage of missing data (7%; Tables 1 and 2). We applied Multiple Imputation using Chained Equations (MICE) to impute missing data in these variables, creating 20 imputed datasets [53]. We used logistic regression models with study group (CoWELL sample, SGP sample), age at study, sex, the scores of the eight HRQOL subscales (PF, RP, BP, GH, VT, SF, RE, MH) and the summary scores (PCS, MCS), and in addition the variables to be imputed, as prediction equations. We performed two different imputations: For the comparison of HRQOL between the CoWELL and the SGP sample (aim 1), we imputed education, and for the identification of determinants in the CoWELL sample only (aim 2), we imputed education, employment status, job type, risk to develop severe COVID-19, own perception about having already had COVID-19, having a person to ask for support, contact with family and friends, frequency of information seeking, and health literacy.

**Table 1:** Characteristics of participants from the CoWELL study (CoWELL sample) and the Swiss general population (SGP sample)

	CoWELL sample *N* = 1581			SGP sample *N* = 1209			*p* value^b^
	Original		Imputed^a^	Original		Imputed^a^	
	*n*	%	%	*n*	%	%	
*Sex*							< 0.001
Male	386	24		507	42		
Female	1195	76		702	58		
*Age at survey*							< 0.001
18–25 years	124	8		92	8		
26–35 years	449	28		164	14		
36–45 years	375	24		231	19		
46–55 years	318	20		278	23		
56–65 years	202	13		232	19		
≥ 66 years	113	7		212	18		
*Highest education*							< 0.001
Compulsory schooling/Vocational training	305	19	20	649	54	57	
Upper secondary education	252	16	16	206	17	18	
University education	972	61	63	288	24	25	
Unknown^c^	52	3	n.a	66	5	n.a	
*Language of questionnaire*							< 0.001
German	1431	91		888	73		
French/Italian	150	9		321	27		
*Employment status*							0.202
Employed/In education	1393	88		868	72		
Other	154	10		310	26		
Unknown^c^	34	2		31	3		
*Childr*en < *14 years in household*							0.002
No	1164	74		950	79		
Yes	417	26		259	21		

	Mean	SD		Mean	SD		*p* value^d^
Age at survey (years)	42.9	13.9		48.7	15.2		< 0.001

*SGP* Swiss general population, *SD* standard deviation

^a^Values derived from Multiple Imputation using Chained Equations (MICE) creating 20 imputed datasets; imputed values are presented in percentages since MICE provides percentages only

^b^p value derived from Chi squared test comparing the CoWELL sample with the SGP sample (based on available original data)

^c^Imputation of missing values in highest education only, because comparison of HRQOL between the CoWELL sample and the SGP sample was adjusted for sex, age at survey, and education

^d^*p* value derived from Student t test comparing the CoWELL sample with the SGP sample

**Table 2:** Characteristics assessed only in participants from the CoWELL study (CoWELL sample)

	CoWell sample *N* = 1581		
	Original		Imputed^a^
	*n*	%	%
*Highest education*			
Compulsory education/Vocational schooling	305	19	20
Upper secondary education	252	16	17
University education	972	61	63
Unknown	52	3	n.a
*Employment status*			
Employed/In education	1393	88	90
Other^b^	154	10	10
Unknown	34	2	n.a
*Job type*			
Health Services	460	29	31
Essential services^c^	204	13	14
Office jobs^d^	703	44	47
Other^e^	109	7	8
Unknown	105	7	n.a
*Living situation*			
Alone	274	17	
Partner	573	36	
Partner and children	456	29	
Parents and/or children	143	9	
Other situation^f^	135	9	
*Physical distancing behaviour*			
Physical distancing	1032	65	
(Self-)isolation	228	14	
No physical distancing/Initial physical distancing	321	20	
*Contact to person with COVID-19*			
No	1302	82	
Yes, assumed/confirmed	279	18	
*Perceived COVID-19*			
No	1408	89	89
Yes	168	11	11
Unknown	5	0.3	n.a
*At risk for severe course of COVID-19*			
No	1331	84	85
Yes	243	15	15
Unknown	7	0.4	n.a
*Having person to ask for support*			
No	47	3	3
Yes	862	55	55
No need for support	646	41	42
Unknown	26	2	n.a
*Contact frequency with family and friends*			
No, not enough contact	292	18	19
Yes, enough/No need for contact	1260	80	81
Unknown	29	2	n.a
*Frequency of information about COVID-19*			
Daily	1016	64	68
Several times per week	333	21	22
Once per week or less	149	9	10
Unknown	83	5	n.a

	Mean	SD
Time since start of pandemic measures (days)^g^	59.2	8.9
Health literacy (score)	42.2	6.2

*SD* standard deviation

^a^Valued derived from Multiple Imputation using Chained Equations (MICE) creating 20 imputed datasets; imputed values are presented in percentages since MICE provides percentages only

^b^Other employment status includes persons who were retired (*n* = 98), managing a household (*n* = 13), seeking for a job (*n* = 24), receiving disability insurance (*n* = 4), or other forms of occupation (*n* = 15)

^c^Essential services include jobs within the areas of agriculture, manufacturing, waste management, construction (*n* = 37); trade, transportation, gastronomy (*n* = 76); education (*n* = 43); social work (*n* = 48)

^d^Office jobs spans the fields of information and communication, finances, insurances, real estate (*n* = 68); scientific and technical activities (*n* = 5); administration (*n* = 414); arts and other service activities (*n* = 216)

^e^Other job type includes persons who were retired (*n* = 51), unemployed (*n* = 46) or not actively working (*n* = 12)

^f^Other living situation includes persons living in a shared apartment (*n* = 101) or other living arrangements (*n* = 34)

^g^Pandemic measures were introduced in Switzerland on 16 March 2020

#### Comparison of HRQOL during (CoWELL sample) and before (SGP sample) the COVID-19 pandemic

For aim 1, we used linear regression models adjusted for sex, age, and education to assess differences in mean physical and mental HRQOL during (CoWELL sample) and before (SGP sample) the COVID-19 pandemic. Positive differences indicate better HRQOL and negative differences poorer HRQOL during compared to before the pandemic. P values for differences were derived from the linear regression models.

#### Investigating determinants for HRQOL in the CoWELL sample

For aim 2, we applied univariable linear regression models to test whether determinants were associated with physical or mental HRQOL and included significant determinants (*p* < 0.05) in multivariable linear regression models. Sex, age, and education were included in the multivariable regression models a priori. For categorical variables, p values were derived from *mi test* (in Stata) to perform joint tests if coefficients are equal to zero for a global effect of the categorical variables on physical or mental HRQOL, respectively. For continuous variables, p values were derived from the linear regression models. We carried out the statistical analyses using Stata 16*.*0 (StataCorp LP, College Station, TX, USA).

## Results

### Samples

The CoWELL sample consisted of 1581 individuals who provided data on HRQOL (76% women, mean age = 43 years (SD = 14 years); Tables 1 and 2, Figure S1). Most participants had a university education (61%) and were employed or in education at time of the survey (88%). Mean time since introduction of restrictive measures in Switzerland (16 March 2020) was 59 days (SD = 9 days).

The SGP sample included 1209 participants (58% women, mean age = 49 years (SD = 15 years); Table 1). More than half (54%) had compulsory education or vocational training as their highest education, and one quarter (24%) had a university education. The majority (72%) were employed or in education at time of the survey.

### Aim 1: comparison of HRQOL during and before the COVID-19 pandemic

**Fig. 1:**
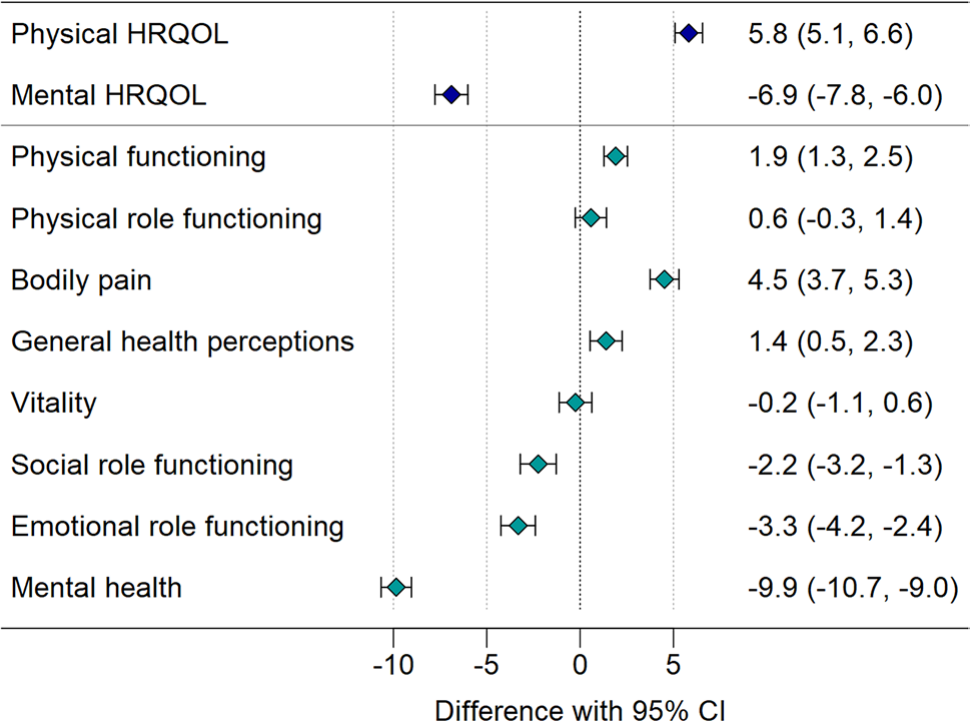
Comparison of HRQOL during (CoWELL sample) and before (Swiss general population sample) the COVID-19 pandemic

Overall, adjusted for sex, age, and education, physical HRQOL was better (PCS, + 5.8, *p* < 0.001) and mental HRQOL was worse (MCS, -6.9, *p* < 0.001) in the CoWELL sample compared to the SGP sample (Fig. 1 and Table S1). More specifically, we found better physical functioning, less bodily pain, and more favourable general health perceptions in the CoWELL sample than in the SGP sample. Social role functioning, emotional role functioning, and mental health were lower in the CoWELL sample than in the SGP sample. Differences between the CoWELL and the SGP sample were particularly high for mental health (MH, −9.9), bodily pain (BP, 4.5), and overall physical and mental HRQOL (PCS, 5.8, and MCS, −6.9), by far exceeding the thresholds for minimally important differences [41].

Figure 1 depicts the differences in physical and mental HRQOL and the eight health domain scales between participants from the CoWELL sample (during the pandemic; *n* = 1581) and the Swiss general population (before the pandemic; *n* = 1209) adjusted for sex, age, and education; positive values indicate better HRQOL and negative values indicate poorer HRQOL during compared to before the COVID-19 pandemic.

### Aim 2: determinants of HRQOL during the COVID-19 pandemic

**Fig. 2:**
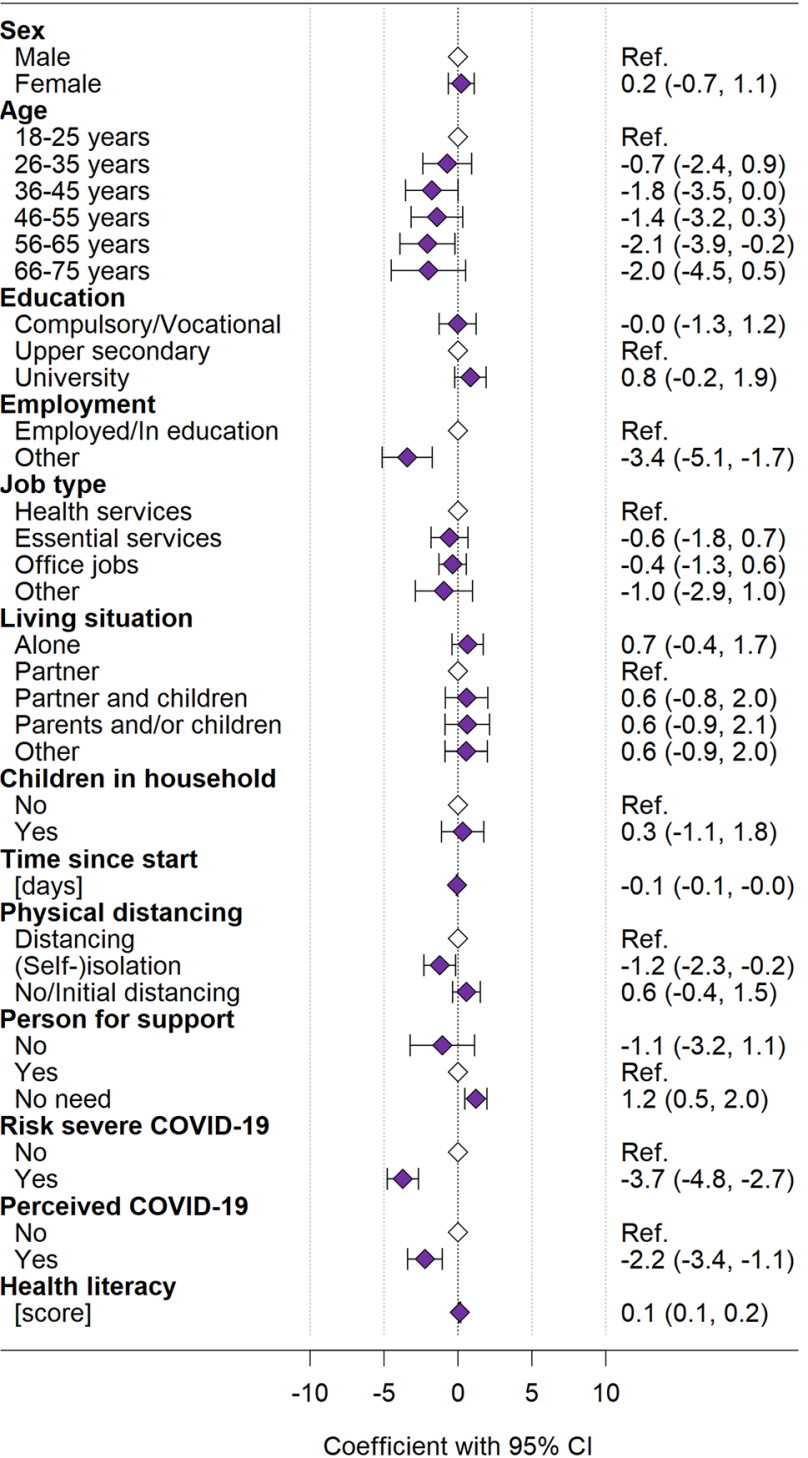
Determinants for physical HRQOL (PCS) in the CoWELL sample from multivariable regression analysis

Physical HRQOL: In the CoWELL sample, persons who responded longer after the start of restrictive measures reported lower physical HRQOL compared to those having responded more closely to the implementation of measures (*p* = 0.002; Fig. 2, Table S2). Persons with better health literacy reported higher physical HRQOL (*p* < 0.001). Persons who were neither employed nor in education reported lower physical HRQOL compared to persons being employed or in education (*p* < 0.001). Physical HRQOL was also lower in persons in (self-)isolation at time of survey (*p* = 0.020), in those at risk to develop severe COVID-19 (*p* < 0.001), and in those perceiving to already have had COVID-19 (*p* < 0.001). Physical HRQOL was better in those who did not need a person to ask for support (*p* = 0.003).

Filled diamonds indicate the coefficients, whiskers indicate the corresponding 95% confidence interval; empty diamonds indicate the reference categories. The multivariable regression analyses included *N* = 1581 participants from the CoWELL sample.

**Fig. 3:**
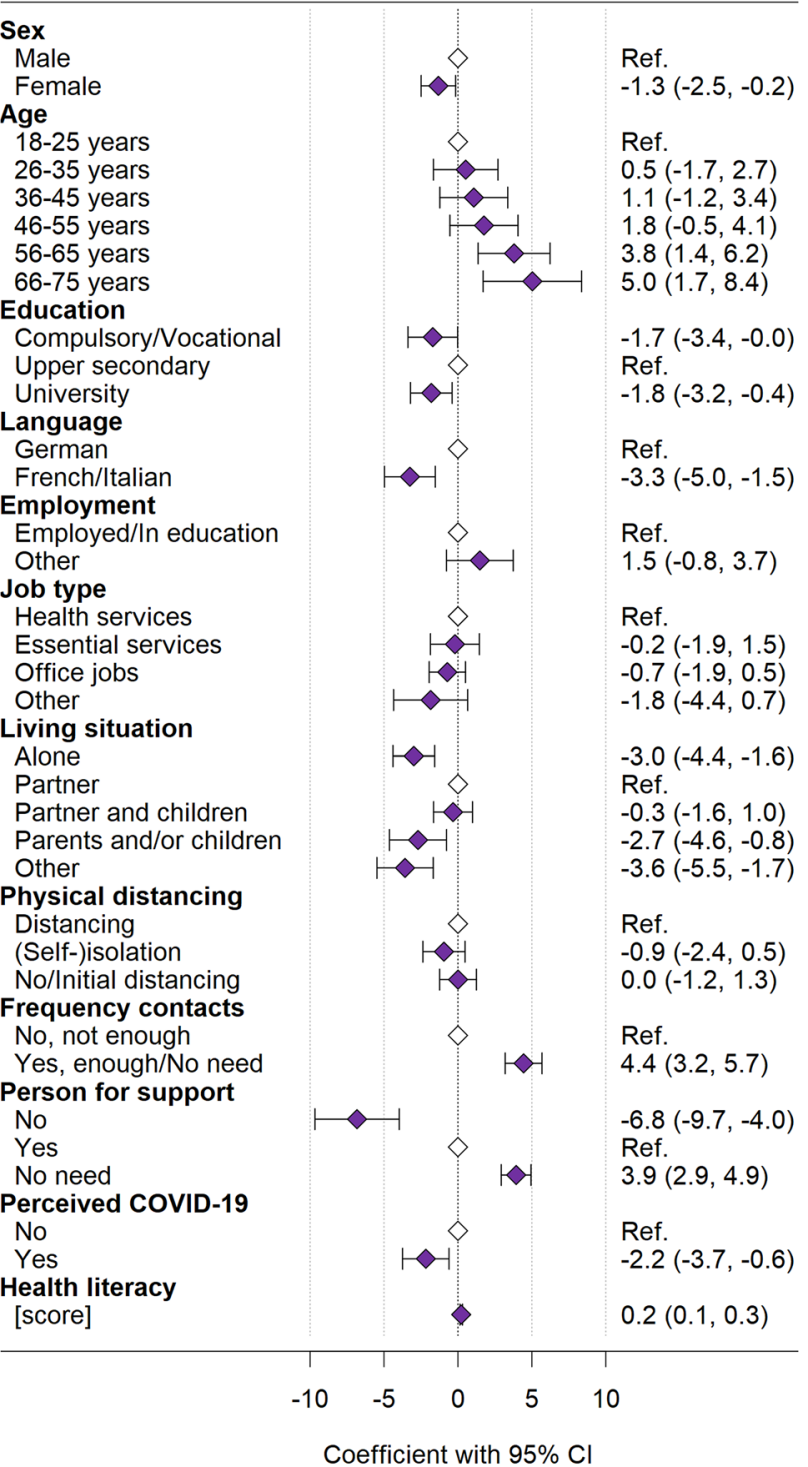
Determinants of mental HRQOL (MCS) in the CoWELL sample from multivariable regression analysis

Mental HRQOL: Older persons (*p* < 0.001) and persons with better health literacy (*p* < 0.001) reported higher mental HRQOL (Fig. 3, Table S3). Mental HRQOL was lower in women (*p* = 0.026), in those with compulsory education/vocational training or with university education compared to those with upper secondary education (*p* = 0.038), in French or Italian speaking persons compared to German speaking persons (*p* < 0.001), and in those living alone, or with their parents and/or children compared to those living only with a partner (*p* < 0.001). Persons reporting not having enough contact with family and friends (*p* < 0.001), not having someone they could ask for support (*p* < 0.001), and those perceiving to already have had COVID-19 (*p* = 0.007) reported lower mental HRQOL.

Filled diamonds indicate the coefficients, whiskers indicate the corresponding 95% confidence interval; empty diamonds indicate the reference categories. The multivariable regression analyses included *N* = 1581 participants from the CoWELL sample.

## Discussion

During the first phase of the pandemic, people from the Swiss general population reported better physical and worse mental HRQOL than before the pandemic in Switzerland. Especially individuals with lower health literacy, who had no support network or who have had a previous COVID-19 experience reported lower physical and mental HRQOL. In contrast, those employed or in education, who practised physical distancing, who were not at high risk for severe COVID-19, and those who responded longer after implementation of restrictive measures reported better physical HRQOL. Better mental HRQOL was associated with being male, being older, having completed upper secondary education, speaking German, living with a partner but without children, and having social contact.

### Physical health-related quality of life

Our study showed that, in Switzerland, physical HRQOL was better in this first phase of the pandemic than before the pandemic. This contrasts with a Japanese study using a representative sample, where physical HRQOL was lower one year after the start of the COVID-19 pandemic [38]. Public health protective measures were different between Switzerland and Japan, and in the Japanese study, more time had passed since the first lockdown than in our study. In Switzerland, individuals were allowed to go outdoors even during periods with strict protective measures. People might have spent their time in the nature doing physical activity as a compensation. Increased physical activity during the early period of the pandemic was associated with improved physical health [54, 55]. Higher perceived physical HRQOL during the first phase of the pandemic might also be due to the comparison of oneself with news reports and pictures of people who were suffering and dying from COVID in the news. This downward comparison might have made individuals feeling better in terms of their own physical health [56]. We also observed that physical HRQOL decreased with more time passed since the implementation of pandemic measures in Switzerland. Discontinuation of commuting to the office and thus increased sedentary behaviour over time could be one reason [57], but also the lack of ergonomic work environments at home might have caused health issues such as back pain [58–60]. We found that individuals who were in isolation at time of the survey or who have already had COVID-19 were at risk for low physical HRQOL, likely because staying at home and the impact being/having been sick with COVID-19 prevented them from physical activity. Individuals at risk for severe course of COVID-19 had lower physical HRQOL than those who had no elevated risk for severe COVID-19. Most of them reported either obesity, cardiovascular health problems, lung diseases or other chronic diseases, which may generally limit their ability to engage in physical activity.

### Mental health-related quality of life

Mental HRQOL was lower during the pandemic than before. This was also observed in Austria [32], in the US [37], and in Japan [38]. The Swiss Corona Stress Study reported increased stress levels during the pandemic and a higher prevalence of depressive symptoms [61]. A systematic review found the lowest point for anxiety and depression after 60 days since the start of the pandemic [62]. The authors also concluded that different populations responded differently to the psychological stress related to the pandemic [62]. Despite an increased risk for severe COVID-19 outcomes in the elderly, the older age groups in our sample reported better mental HRQOL than younger people. This might be related to increased resilience at older age [46, 63]. COVID-19 was more prevalent in the Italian and French speaking part of Switzerland during the time of our study [64]. Persons from these regions reported lower mental HRQOL before [46] and during the pandemic.

Changed daily routines and the massive cut on social life might have contributed to lower mental HRQOL. Increased parental duties with suspended childcare and home schooling might have further impaired mental HRQOL. We found lower mental HRQOL in persons living alone and persons living with parents and/or children in the same household, but not in participants with partner and children in the same household. Previous studies from the US, Australia and Italy found also a relationship between more childcare and lower mental health or more psychological distress [37, 65, 66]. A systematic review found that parents experienced high stress, anxiety, and financial burden during pandemics [67]. Mental HRQOL was lower in individuals who felt socially isolated and in those who had no one to ask for support. Loneliness was a risk factor for decreased mental well-being and life satisfaction before and during the pandemic [68–70], and decreased social interactions during the pandemic were associated with reduced quality of life and increased depression [71]. We found that individuals who have already had COVID-19 had a lower mental HRQOL, possibly because some of them had persistent symptoms. Similarly, in US patients, the burden of symptoms persistent for at least six months after mild COVID-19 was associated with low mental HRQOL and psychological distress [72].

### Health literacy and health-related quality of life

Low health literacy was a risk factor for both, low physical and low mental HRQOL during the pandemic in Switzerland. This was similar in a large systematic review and meta-analysis in populations with chronic diseases [73]. A study among the Japanese general population found that individuals with higher health literacy before the pandemic reported a smaller decline in physical and mental HRQOL during the pandemic [38]. Health literate individuals have better capacities to access, understand and interpret health information, and to use this knowledge to make decisions for prevention and actions in health issues. This ability may help them to better cope with a major health crisis, such as the COVID-19 pandemic [74].

### Limitations and strengths

Our study has the limitation of using a convenience sample which overrepresents women and well-educated participants and preventing us from being able to calculate a response rate. This is due to biases arising from our recruitment strategy [75], but alternative recruitment designs were not attainable to us. Swiss legislation did not permit to re-contact participants from the *SGP sample* and it was not possible to get another representative sample from SFSO. We have adjusted for age, sex, and education in all our regression analyses to account for differences in these variables between the CoWELL and the SGP sample. Yet, conclusions largely apply to well-educated women and might not be similar for the Swiss general population. Our study has the strengths of being able to compare HRQOL from before to during the pandemic, to include a large number of participants, and to use a wide range of traditional socio-demographic, socio-economic, and COVID-19-related covariables. This allowed us, with good statistical power, to disentangle the pandemic-specific impact on HRQOL. The study started two months after introducing preventive measures in Switzerland, which allowed to measure direct pandemic effects on HRQOL, when people had not yet fully adapted to pandemic routines. We assessed HRQOL with the SF-36, a widely used psychometrically valid instrument [44], and used recent SF-36 normative data from Switzerland, which ensured a recent and appropriate comparison sample for HRQOL in Switzerland.

## Conclusions

During the pandemic, physical HRQOL was better and mental HRQOL worse than before the pandemic in Switzerland. In addition to the established determinants of HRQOL, individuals with a COVID-19 experience, less interpersonal support, and with lower health literacy are especially prone to impaired HRQOL during the COVID-19 pandemic.

## Supplementary Information

Below is the link to the electronic supplementary material.

Supplementary file1 (DOCX 125 KB)

## Data Availability

The data that support the information of this manuscript were accessed on secured servers of the Faculty of Health Sciences and Medicine at the University of Lucerne. Individual-level sensitive data can only be made available for researchers who fulfil the respective legal requirements. All data requests should be communicated to the corresponding author.

## Abbreviations

SGP: Swiss general population
SNSF: Swiss National Science Foundation
COVID-19: Corona Virus Disease 2019
b: Beta coefficient
BP: Bodily pain
CI: Confidence interval
GH: General health
HRQOL: Health-related quality of life
MCS: Mental Component Summary
MH: Mental health
PCS: Physical Component Summary
PF: Physical functioning
RE: Emotional role functioning
RP: Physical role functioning
SF: Social role functioning
SFSO: Swiss Federal Statistical Office
SF-36v2: Short Form-36 version 2
VT: Vitality

## References

[1] Hauser A, Counotte MJ, Margossian CC, Konstantinoudis G, Low N, Althaus CL (2020). Estimation of SARS-CoV-2 mortality during the early stages of an epidemic: A modeling study in Hubei, China, and six regions in Europe. PLoS Medicine.

[2] World Health Organization (WHO). (2022). WHO Coronavirus (COVID-19) Dashboard. Retrieved from https://web.archive.org/web/20220101025644/https://covid19.who.int/

[3] Federal Office of Public Health (FOPH). (2020). New coronavirus: Federal government measures. https://web.archive.org/web/20200318221654/https://www.bag.admin.ch/bag/en/home/krankheiten/ausbrueche-epidemien-pandemien/aktuelle-ausbrueche-epidemien/novel-cov/massnahmen-des-bundes.html.

[4] Lemaitre JC, Perez-Saez J, Azman AS, Rinaldo A, Fellay J (2020). Assessing the impact of non-pharmaceutical interventions on SARS-CoV-2 transmission in Switzerland. Swiss Medical Weekly.

[5] Ware JE, Gandek B (1998). Overview of the SF-36 health survey and the international quality of life assessment (IQOLA) Project. Journal of Clinical Epidemiology.

[6] Reeve BB, Mitchell SA, Dueck AC, Basch E, Cella D, Reilly CM (2014). Recommended patient-reported core set of symptoms to measure in adult cancer treatment trials. J Natl Cancer Inst.

[7] Fromme EK, Eilers KM, Mori M, Hsieh YC, Beer TM (2004). How accurate is clinician reporting of chemotherapy adverse effects? A comparison with patient-reported symptoms from the quality-of-life questionnaire C30. Journal of Clinical Oncology.

[8] Moons P, Goossens E, Luyckx K, Kovacs AH, Andresen B, Moon JR (2021). The COVID-19 pandemic as experienced by adults with congenital heart disease from Belgium, Norway, and South Korea: Impact on life domains, patient-reported outcomes, and experiences with care. Eur J Cardiovasc Nurs.

[9] Sahin S, Karsidag S, Cinar N, Ates MF, Demir S, Eren F (2021). The impact of the COVID-19 lockdown on the quality of life in chronic neurological diseases: The results of a COVQoL-CND study. European Neurology.

[10] Greco F, Altieri VM, Esperto F, Mirone V, Scarpa RM (2021). Impact of COVID-19 pandemic on health-related quality of life in uro-oncologic patients: What should we wait for?. Clinical Genitourinary Cancer.

[11] Tan HQM, Pendolino AL, Andrews PJ, Choi D (2022). Prevalence of olfactory dysfunction and quality of life in hospitalised patients 1 year after SARS-CoV-2 infection: A cohort study. British Medical Journal Open.

[12] Egede LE, Walker RJ, Dawson AZ, Zosel A, Bhandari S, Nagavally S (2022). Short-term impact of COVID-19 on quality of life, perceived stress, and serious psychological distress in an adult population in the Midwest United States. Qual Life Res.

[13] Poudel AN, Zhu S, Cooper N, Roderick P, Alwan N, Tarrant C (2021). Impact of Covid-19 on health-related quality of life of patients: A structured review. PLoS ONE.

[14] Lapin B, Katzan IL (2022). Health-related quality of life mildly affected following COVID-19: A retrospective pre-post cohort study with a propensity score-matched control group. J Gen Intern Med.

[15] Ferreira LN, Pereira LN, da Fé BM, Ilchuk K (2021). Quality of life under the COVID-19 quarantine. Quality of Life Research.

[16] Vu MQ, Tran TTP, Hoang TA, Khuong LQ, Hoang MV (2020). Health-related quality of life of the Vietnamese during the COVID-19 pandemic. PLoS ONE.

[17] Duan Y, Peiris D, Yang M, Liang W, Baker JS, Hu C (2021). Lifestyle behaviors and quality of life among older adults after the first wave of the COVID-19 pandemic in Hubei China. Frontiers in Public Health.

[18] Colucci E, Nadeau S, Higgins J, Kehayia E, Poldma T, Saj A (2022). COVID-19 lockdowns’ effects on the quality of life, perceived health and well-being of healthy elderly individuals: A longitudinal comparison of pre-lockdown and lockdown states of well-being. Archives of Gerontology and Geriatrics.

[19] Bringolf-Isler B, Hänggi J, Kayser B, Suggs LS, Dössegger A, Probst-Hensch N (2021). COVID-19 pandemic and health related quality of life in primary school children in Switzerland: A repeated cross-sectional study. Swiss Medical Weekly.

[20] Ravens-Sieberer U, Kaman A, Erhart M, Devine J, Schlack R, Otto C (2021). Impact of the COVID-19 pandemic on quality of life and mental health in children and adolescents in Germany. Eur Child Adolesc Psychiatry.

[21] Platt B, Danzer V, Schulte-Körne G (2021). Critical reflections on the manuscript “Impact of the COVID-19 pandemic on quality of life and mental health in children and adolescents in Germany” published in ECAP on January 25th. European Child and Adolescent Psychiatry.

[22] Dragun R, Veček NN, Marendić M, Pribisalić A, Đivić G, Cena H (2020). Have lifestyle habits and psychological well-being changed among adolescents and medical students due to COVID-19 lockdown in Croatia?. Nutrients.

[23] Riiser K, Helseth S, Haraldstad K, Torbjørnsen A, Richardsen KR (2020). Adolescents’ health literacy, health protective measures, and health-related quality of life during the Covid-19 pandemic. PLoS ONE.

[24] Epifanio MS, Andrei F, Mancini G, Agostini F, Piombo MA, Spicuzza V (2021). The impact of COVID-19 pandemic and lockdown measures on quality of life among Italian general population. J Clin Med.

[25] van Ballegooijen H, Goossens L, Bruin RH, Michels R, Krol M (2021). Concerns, quality of life, access to care and productivity of the general population during the first 8 weeks of the coronavirus lockdown in Belgium and the Netherlands. BMC Health Services Research.

[26] Candeias A, Galindo E, Stueck M, Portelada A, Knietzsch J (2021). Psychological adjustment, quality of life and well-being in a German and Portuguese adult population during COVID-19 pandemics crisis. Frontiers in Psychology.

[27] Long D, Haagsma JA, Janssen MF, Yfantopoulos JN, Lubetkin EI, Bonsel GJ (2021). Health-related quality of life and mental well-being of healthy and diseased persons in 8 countries: Does stringency of government response against early COVID-19 matter?. SSM Popul Health.

[28] Hansel TC, Saltzman LY, Melton PA, Clark TL, Bordnick PS (2022). COVID-19 behavioral health and quality of life. Science and Reports.

[29] Geirdal A, Ruffolo M, Leung J, Thygesen H, Price D, Bonsaksen T (2021). Mental health, quality of life, wellbeing, loneliness and use of social media in a time of social distancing during the COVID-19 outbreak: A cross-country comparative study. J Ment Health.

[30] Ikeda T, Igarashi A, Odani S, Murakami M, Tabuchi T (2021). Health-related quality of life during COVID-19 pandemic: Assessing impacts of job loss and financial support programs in Japan. Applied Research in Quality Life.

[31] Azizi A, Achak D, Aboudi K, Saad E, Nejjari C, Nouira Y (2020). Health-related quality of life and behavior-related lifestyle changes due to the COVID-19 home confinement: Dataset from a Moroccan sample. Data in Brief.

[32] Pieh C, Budimir S, Delgadillo J, Barkham M, Fontaine JRJ, Probst T (2021). Mental health during COVID-19 lockdown in the United Kingdom. Psychosomatic Medicine.

[33] Herrera MS, Elgueta R, Fernández MB, Giacoman C, Leal D, Marshall P (2021). A longitudinal study monitoring the quality of life in a national cohort of older adults in Chile before and during the COVID-19 outbreak. BMC Geriatrics.

[34] Barone Gibbs B, Kline CE, Huber KA, Paley JL, Perera S (2021). Covid-19 shelter-at-home and work, lifestyle and well-being in desk workers. Occupational Medicine (London).

[35] Koinig KA, Arnold C, Lehmann J, Giesinger J, Köck S, Willenbacher W (2021). The cancer patient’s perspective of COVID-19-induced distress—A cross-sectional study and a longitudinal comparison of HRQOL assessed before and during the pandemic. Cancer Medicine.

[36] Pieh C, Budimir S, Probst T (2020). The effect of age, gender, income, work, and physical activity on mental health during coronavirus disease (COVID-19) lockdown in Austria. Journal of Psychosomatic Research.

[37] Romm KF, Patterson B, Wysota CN, Wang Y, Berg CJ (2022). Predictors of negative psychosocial and health behavior impact of COVID-19 among young adults. Health Education Research.

[38] Ishikawa H, Kato M, Kiuchi T (2021). Declines in health literacy and health-related quality of life during the COVID-19 pandemic: A longitudinal study of the Japanese general population. BMC Public Health.

[39] Our World in Data. (2020). COVID-19 Stringency Index on 1 June 2020. Retrieved from https://ourworldindata.org/grapher/covid-stringency-index?time=2020-06-01

[40] Ware JE, Sherbourne CD (1992). The MOS 36-item short-form health survey (SF-36) I: Conceptual framework and item selection. Med Care.

[41] Maruish ME (2011). User’s manual for the SF-36v2 health survey.

[42] Morfeld M, Kirchberger I, Bullinger M (2011). SF-36 Fragebogen zum Gesundheitszustand Deutsche Version des Short Form-36 Health Survey 2, ergänzte und überarbeitete Auflage Manual.

[43] Wagner AK, Gandek B, Aaronson NK, Acquadro C, Alonso J, Apolone G (1998). Cross-cultural comparisons of the content of SF-36 translations across 10 countries: Results from the IQOLA Project: International quality of life assessment. Journal of Clinical Epidemiology.

[44] Ware JE, Kosinski M, Bjorner JB, Turner-Bowker DM, Gandek B, Maruish ME (2008). SF-36v2® health survey: Administration guide for clinical trial investigators.

[45] Ware JE (1993). SF-36 health survey manual & interpretation guide.

[46] Roser K, Mader L, Baenziger J, Sommer G, Kuehni CE, Michel G (2019). Health-related quality of life in Switzerland: Normative data for the SF-36v2 questionnaire. Quality of Life Research.

[47] Bundesamt für Statistik. (2019). Durchschnittliche Körpergrösse (in cm)

[48] Swiss Federal Statistical Office (2008). General classification of economic activities.

[49] Finbråten HS, Wilde-Larsson B, Nordström G, Pettersen KS, Trollvik A, Guttersrud Ø (2018). Establishing the HLS-Q12 short version of the European health literacy survey questionnaire: Latent trait analyses applying Rasch modelling and confirmatory factor analysis. BMC Health Services Research.

[50] Sørensen K, Van den Broucke S, Pelikan JM, Fullam J, Doyle G, Slonska Z (2013). Measuring health literacy in populations: Illuminating the design and development process of the European health literacy survey questionnaire (HLS-EU-Q). BMC Public Health.

[51] Clinical Group of the Swiss National COVID-19 Science Task Force. (2020). Risk factors for severe manifestations of SARS-CoV-2 infection. Retrieved from https://sciencetaskforce.ch/en/policy-brief/risk-factors-for-severe-manifestations-of-sars-cov-2-infection/

[52] Federal Office of Public Health (FOPH). Coronavirus: Measures and ordinances. Retrieved 20 June 2022, from https://www.bag.admin.ch/bag/en/home/krankheiten/ausbrueche-epidemien-pandemien/aktuelle-ausbrueche-epidemien/novel-cov/massnahmen-des-bundes.html

[53] StataCorp. (2021). Stata multiple-imputation reference manual release 17. Retrieved from https://www.stata.com/manuals/mi

[54] Refle J-E, Voorpostel M, Lebert F, Kuhn U, Klaas HS, Ryser V-A, Dasoki N, Monsch G-A, Antal E, Tillmann R (2020). First results of the Swiss household panel—Covid-19 study.

[55] Cheval B, Sivaramakrishnan H, Maltagliati S, Fessler L, Forestier C, Sarrazin P (2021). Relationships between changes in self-reported physical activity, sedentary behaviour and health during the coronavirus (COVID-19) pandemic in France and Switzerland. Journal of Sports Sciences.

[56] Litt DM, Fairlie AM, Lewis MA, Stock ML (2020). Health correlates and consequences of social comparison.

[57] Stockwell S, Trott M, Tully M, Shin J, Barnett Y, Butler L (2021). Changes in physical activity and sedentary behaviours from before to during the COVID-19 pandemic lockdown: A systematic review. BMJ Open Sport & Exercise Medicine.

[58] Dockrell S, Culleton-Quinn E (2023). Remote working during the COVID-19 pandemic: Computer-related musculoskeletal symptoms in university staff. Work.

[59] Gerding T, Syck M, Daniel D, Naylor J, Kotowski SE, Gillespie GL (2021). An assessment of ergonomic issues in the home offices of university employees sent home due to the COVID-19 pandemic. Work.

[60] Wütschert MS, Romano-Pereira D, Suter L, Schulze H, Elfering A (2022). A systematic review of working conditions and occupational health in home office. Work.

[61] de Quervain, D., Aerni, A., Amini, E., et al. (2020). The Swiss corona stress study

[62] Salanti G, Peter N, Tonia T, Holloway A, White IR, Darwish L (2022). The impact of the COVID-19 pandemic and associated control measures on the mental health of the general population: A systematic review and dose-response meta-analysis. Annals of Internal Medicine.

[63] Lim XY, Yap AC, Mahendran R, Yu J (2021). The interplay between anxiety, fear, protective behaviors, compassion, and resilience among older adults during a COVID-19 lockdown: A structural equation modeling study. Translational Behaviour Medicine.

[64] Federal Office of Public Health FOPH. (2020). Geographic distribution over time. Retrieved from https://www.covid19.admin.ch/en/epidemiologic/case/d/geo-regions?geoDate=2020-03-12

[65] Calear AL, McCallum S, Morse AR, Banfield M, Gulliver A, Cherbuin N (2022). Psychosocial impacts of home-schooling on parents and caregivers during the COVID-19 pandemic. BMC Public Health.

[66] Marchetti D, Fontanesi L, Mazza C, Di Giandomenico S, Roma P, Verrocchio MC (2020). Parenting-related exhaustion during the Italian COVID-19 lockdown. Journal of Pediatric Psychology.

[67] Fong VC, Iarocci G (2020). Child and family outcomes following pandemics: A systematic review and recommendations on COVID-19 policies. Journal of Pediatric Psychology.

[68] Clair R, Gordon M, Kroon M, Reilly C (2021). The effects of social isolation on well-being and life satisfaction during pandemic. Humanities and Social Sciences Communications.

[69] Leigh-Hunt N, Bagguley D, Bash K, Turner V, Turnbull S, Valtorta N (2017). An overview of systematic reviews on the public health consequences of social isolation and loneliness. Public Health.

[70] Rumas R, Shamblaw AL, Jagtap S, Best MW (2021). Predictors and consequences of loneliness during the COVID-19 pandemic. Psychiatry Research.

[71] Lebrasseur A, Fortin-Bédard N, Lettre J, Raymond E, Bussières EL, Lapierre N (2021). Impact of the COVID-19 pandemic on older adults: rapid review. JMIR Aging.

[72] Han JH, Womack KN, Tenforde MW, Files DC, Gibbs KW, Shapiro NI (2022). Associations between persistent symptoms after mild COVID-19 and long-term health status, quality of life, and psychological distress. Influenza and Other Respiratory Viruses.

[73] Zheng M, Jin H, Shi N, Duan C, Wang D, Yu X (2018). The relationship between health literacy and quality of life: A systematic review and meta-analysis. Health and Quality of Life Outcomes.

[74] Ilic A, Roser K, Sommer G, Baenziger J, Mitter VR, Mader L (2022). COVID-19 information-seeking, health literacy, and worry and anxiety during the early stage of the pandemic in Switzerland: A cross-sectional study. International Journal of Public Health.

[75] Stratton SJ (2021). Population research: Convenience sampling strategies. Prehospital and Disaster Medicine.

