# Supplementary material for: Health-related quality of life before and during the COVID-19 pandemic in Switzerland: a cross-sectional study
Source: Qual Life Res. 2023 Apr 21;32(9):2695–706. doi: 10.1007/s11136-023-03414-0 (PMC10119820; doi:10.1007/s11136-023-03414-0)

**Supplementary Material**

**Health-related quality of life before and during the COVID-19 pandemic in Switzerland**

Katharina Roser^1^, Julia Baenziger^1,2,3^, Anica Ilic^1^, Vera R. Mitter^4^, Luzius Mader^5,6^, Daniela Dyntar^1,7^, Gisela Michel^1^, and Grit Sommer^5,8,9^

**Affiliations**

^1^ Faculty of Health Sciences and Medicine, University of Lucerne, Lucerne, Switzerland

^2^ Heart Centre for Children, The Sydney Children’s Hospitals Network, Sydney, NSW, Australia.

^3^ Center for Heart Disease and Mental Health, Heart Institute and the Division of Behavioral Medicine and Clinical Psychology, Cincinnati Children’s Hospital Medical Center and the Department of Pediatrics, University of Cincinnati College of Medicine, Cincinnati, OH, United States.

^4^ Department of Gynaecology, Bern University Hospital, University of Bern, Bern, Switzerland

^5^ Institute of Social and Preventive Medicine, University of Bern, Bern, Switzerland

^6^ Cancer Registry Bern-Solothurn, University of Bern, Murtenstrasse 31, 3008 Bern

^7^ Swiss Childhood Cancer Registry, University of Bern, Bern, Switzerland

^8^ Department for BioMedical Research, University of Bern, Bern, Switzerland

^9^ Pediatric Endocrinology, Diabetology and Metabolism, Department of Pediatrics, Inselspital, Bern University Hospital, University of Bern, Bern, Switzerland

**ORCID**

Katharina Roser: 0000-0001-5253-3333

Julia Baenziger: 0000-0001-9121-4423

Anica Ilic: 0000-0001-6863-6441

Vera R. Mitter: 0000-0002-1483-5020

Luzius Mader: 0000-0001-5613-4356

Daniela Dyntar: 0000-0001-9921-1462

Gisela Michel: 0000-0002-9589-0928

Grit Sommer: 0000-0002-4205-7932

**Corresponding author**

Katharina Roser, Faculty of Health Sciences and Medicine, University of Lucerne, Lucerne, Switzerland; katharina.roser@unilu.ch, Tel +41 41 229 59 56

**Table S1:** Comparison of HRQOL during (CoWELL sample; n=1581) and before (Swiss general population (SGP) sample; n=1209) the COVID-19 pandemic; differences between participants from the CoWELL and the SGP sample and unadjusted T-scores of each sample for physical HRQOL (PCS) and mental HRQOL (MCS) and the eight health domain subscales.

|  |  |  |  | **CoWELL sample** | | **SGP sample** | |
| --- | --- | --- | --- | --- | --- | --- | --- |
| **Scale or subscale** | **Difference^a^** | **95% CI difference^a^** | **p value^a^** | **Mean T‑Score^b^** | **95% CI T‑score^b^** | **Mean T‑score^b^** | **95% CI T‑score^b^** |
| Physical HRQOL (PCS) | 5.82 | (5.08, 6.56) | **<0.001** | 56.9 | (56.5, 57.3) | 50.0 | (49.4, 50.6) |
| Mental HRQOL (MCS) | -6.89 | (-7.78, -6.01) | **<0.001** | 42.0 | (41.5, 42.5) | 50.0 | (49.4, 50.6) |
| Physical functioning (PF) | 1.91 | (1.28, 2.54) | **<0.001** | 53.0 | (52.7, 53.2) | 50.0 | (49.5, 50.6) |
| Physical role functioning (RP) | 0.58 | (-0.25, 1.42) | 0.173 | 51.1 | (50.6, 51.6) | 50.0 | (49.4, 50.5) |
| Bodily pain (BP) | 4.52 | (3.75, 5.29) | **<0.001** | 54.7 | (54.3, 55.1) | 50.0 | (49.4, 50.6) |
| General health perceptions (GH) | 1.39 | (0.53, 2.25) | **0.002** | 52.0 | (51.5, 52.5) | 49.9 | (49.3, 50.5) |
| Vitality (VT) | -0.25 | (-1.12, 0.63) | 0.577 | 48.5 | (48.0, 49.0) | 49.9 | (49.3, 50.5) |
| Social role functioning (SF) | -2.24 | (-3.21, -1.28) | **<0.001** | 47.5 | (46.9, 48.0) | 49.9 | (49.3, 50.5) |
| Emotional role functioning (RE) | -3.32 | (-4.23, -2.40) | **<0.001** | 46.1 | (45.5, 46.7) | 49.8 | (49.3, 50.4) |
| Mental health (MH) | -9.85 | (-10.66, -9.04) | **<0.001** | 39.2 | (38.8, 39.6) | 49.9 | (49.3, 50.5) |

**Comments:** Difference: Positive values indicate better HRQOL and negative values poorer HRQOL during (CoWELL sample) compared to before (SGP sample) the COVID-19 pandemic; p values<0.05 are indicated in bold.

**Abbreviations**: BP, Bodily pain; CI, confidence interval; COVID-19, corona virus disease 2019; GH, General health perceptions; HRQOL, health-related quality of life; MCS, Mental Component Summary; MH, Mental health; PCS, Physical Component Summary; PF, Physical functioning; RE, Emotional role functioning; RP, Physical role functioning; SF, Social role functioning; SGP, Swiss general population; VT, Vitality.

**Footnotes:**

^a^ Derived from linear regression models adjusted for sex, age, and education.

^b^ Unadjusted mean and 95%CI T-Scores.

**Table S2:** Results from univariable and multivariable linear regression models for physical HRQOL (PCS) in the CoWELL sample (n=1581).

|  | **Univariable** | | | |  | **Multivariable** | | | |
| --- | --- | --- | --- | --- | --- | --- | --- | --- | --- |
|  | **Coeff.** | **95% CI** | | **p value^a^** |  | **Coeff.** | **95% CI** | | **p value^a^** |
| **Sex** |  |  |  | 0.059 |  |  |  |  | 0.622 |
| Male | ref |  |  |  |  | ref |  |  |  |
| Female | 0.8 | -0.03 | 1.7 |  |  | 0.2 | -0.7 | 1.1 |  |
| **Age at survey** |  |  |  | **<0.001** |  |  |  |  | 0.128 |
| 18-25 years | ref |  |  |  |  | ref |  |  |  |
| 26-35 years | -0.8 | -2.3 | 0.6 |  |  | -0.7 | -2.4 | 0.9 |  |
| 36-45 years | -1.8 | -3.3 | -0.2 |  |  | -1.8 | -3.5 | 0.004 |  |
| 46-55 years | -1.5 | -3.1 | 0.1 |  |  | -1.4 | -3.2 | 0.3 |  |
| 56-65 years | -3.0 | -4.7 | -1.3 |  |  | -2.1 | -3.9 | -0.2 |  |
| ≥66 years | -6.8 | -8.7 | -4.9 |  |  | -2.0 | -4.5 | 0.5 |  |
| **Highest education** |  |  |  | **<0.001** |  |  |  |  | 0.134 |
| Compulsory schooling/Vocational training | -0.3 | -1.6 | 1.0 |  |  | -0.02 | -1.3 | 1.2 |  |
| Upper secondary education | ref |  |  |  |  | ref |  |  |  |
| University education | 1.4 | 0.4 | 2.5 |  |  | 0.8 | -0.2 | 1.9 |  |
| **Language of questionnaire** |  |  |  |  |  |  |  |  |  |
| German | ref |  |  | 0.154 |  |  |  |  |  |
| French/Italian | -0.9 | -2.2 | 0.4 |  |  |  |  |  |  |
| **Children <14 years in household** | |  |  | **0.030** |  |  |  |  | 0.661 |
| No | ref |  |  |  |  | ref |  |  |  |
| Yes | 1.0 | 0.1 | 1.8 |  |  | 0.3 | -1.1 | 1.8 |  |
| **Employment status** |  |  |  | **<0.001** |  |  |  |  | **<0.001** |
| Employed/In education | ref |  |  |  |  | ref |  |  |  |
| Other^b^ | -5.3 | -6.5 | -4.0 |  |  | -3.4 | -5.1 | -1.7 |  |
| **Job type** |  |  |  | **<0.001** |  |  |  |  | 0.691 |
| Health Services | ref |  |  |  |  | ref |  |  |  |
| Essential services^c^ | -1.3 | -2.6 | -0.1 |  |  | -0.6 | -1.8 | 0.7 |  |
| Office jobs^d^ | -0.5 | -1.4 | 0.4 |  |  | -0.4 | -1.3 | 0.6 |  |
| Other^e^ | -3.8 | -5.4 | -2.3 |  |  | -1.0 | -2.9 | 1.0 |  |
| **Living situation** |  |  |  | **0.026** |  |  |  |  | 0.741 |
| Alone | 0.5 | -0.6 | 1.6 |  |  | 0.7 | -0.4 | 1.7 |  |
| Partner | ref |  |  |  |  | ref |  |  |  |
| Partner and children | 1.3 | 0.4 | 2.3 |  |  | 0.6 | -0.8 | 2.0 |  |
| Parents and/or children | 1.0 | -0.4 | 2.4 |  |  | 0.6 | -0.9 | 2.1 |  |
| Other situation^f^ | 1.8 | 0.4 | 3.3 |  |  | 0.6 | -0.9 | 2.0 |  |
| **Time since start of pandemic measures (days)** | -0.1 | -0.1 | -0.02 | **0.005** |  | -0.1 | -0.1 | -0.02 | **0.002** |
| **Physical distancing behaviour** |  |  |  | **<0.001** |  |  |  |  | **0.020** |
| Physical distancing | ref |  |  |  |  | ref |  |  |  |
| (Self-)isolation | -2.0 | -3.1 | -0.9 |  |  | -1.2 | -2.3 | -0.2 |  |
| No physical distancing/Initial physical distancing | 0.7 | -0.3 | 1.6 |  |  | 0.6 | -0.4 | 1.5 |  |
| **Contact to person with COVID-19** |  |  |  | 0.384 |  |  |  |  |  |
| No | ref |  |  |  |  |  |  |  |  |
| Yes, assumed/confirmed | 0.4 | -0.6 | 1.4 |  |  |  |  |  |  |
| **Perceived COVID-19** |  |  |  | **0.006** |  |  |  |  | **<0.001** |
| No | ref |  |  |  |  | ref |  |  |  |
| Yes | -1.7 | -2.9 | -0.5 |  |  | -2.2 | -3.4 | -1.1 |  |
| **At risk for severe course of COVID-19** |  |  |  | **<0.001** |  |  |  |  | **<0.001** |
| No | ref |  |  |  |  | ref |  |  |  |
| Yes | -4.7 | -5.7 | -3.7 |  |  | -3.7 | -4.8 | -2.7 |  |
|  |  |  |  |  |  |  |  |  |  |
| **Having person to ask for support** |  |  |  | **0.005** |  |  |  |  | **0.003** |
| No | -1.4 | -3.7 | 0.9 |  |  | -1.1 | -3.2 | 1.1 |  |
| Yes | ref |  |  |  |  | ref |  |  |  |
| No need for support | 1.1 | 0.4 | 1.9 |  |  | 1.2 | 0.5 | 2.0 |  |
| **Contact frequency with family and friends** |  |  |  | 0.201 |  |  |  |  |  |
| No, not enough contact | ref |  |  |  |  |  |  |  |  |
| Yes, enough/No need for contact | -0.6 | -1.6 | 0.3 |  |  |  |  |  |  |
| **Frequency of information about COVID-19** |  |  |  |  |  |  |  |  |  |
| Daily | ref |  |  | 0.802 |  |  |  |  |  |
| Several times per week | 0.3 | -0.6 | 1.3 |  |  |  |  |  |  |
| Once per week or less | 0.02 | -1.3 | 1.3 |  |  |  |  |  |  |
| **Health literacy (score)** | 0.2 | 0.1 | 0.2 | **<0.001** |  | 0.1 | 0.1 | 0.2 | **<0.001** |

**Comments:** Higher scores of the PCS indicate better physical HRQOL. Multivariable linear regression models included sex, age at survey, and highest education level regardless of their significance in the univariable regression model, because we assumed *a priori* that these variables were important predictors of HRQOL. p values<0.05 are indicated in bold.

**Abbreviations:** CI, confidence interval; Coeff, coefficient; COVID-19, corona virus disease 2019; HRQOL, health-related quality of life; PCS, Physical Component Summary; ref, reference category.

**Footnotes:**

^a^ P values derived from *mi test* (in Stata) to perform joint tests that coefficients are equal to zero for a global effect of the categorial variable on physical HRQOL (PCS).

^b^ Other employment status includes persons who were retired, managing a household, seeking for a job, receiving disability insurance, or other forms of occupation.

^c^ Essential services include jobs within the areas of agriculture, manufacturing, waste management, construction; trade, transportation, gastronomy; education; social work.

^d^ Office jobs spans the fields of information and communication, finances, insurances, real estate; scientific and technical activities; administration; arts and other service activities.

^e^ Other jobe type includes persons who were retired, unemployed or not actively working.

^f^ Other living situation includes persons living in a shared apartment or other living arrangements.

**Table S3:** Results from univariable and multivariable linear regression models for mental HRQOL (MCS) in the CoWELL sample (n=1581).

|  | **Univariable** | | | |  | **Multivariable** | | | |
| --- | --- | --- | --- | --- | --- | --- | --- | --- | --- |
|  | **Coeff.** | **95% CI** | | **p value^a^** |  | **Coeff.** | **95% CI** | | **p value^a^** |
| **Sex** |  |  |  | **0.005** |  |  |  |  | **0.026** |
| Male | ref |  |  |  |  | ref |  |  |  |
| Female | -1.8 | -3.0 | -0.5 |  |  | -1.3 | -2.5 | -0.2 |  |
| **Age at survey (years)** |  |  |  |  |  |  |  |  |  |
| 18-25 years | ref |  |  | **<0.001** |  | ref |  |  | **<0.001** |
| 26-35 years | 2.1 | -0.01 | 4.1 |  |  | 0.5 | -1.7 | 2.7 |  |
| 36-45 years | 3.2 | 1.1 | 5.4 |  |  | 1.1 | -1.2 | 3.4 |  |
| 46-55 years | 5.0 | 2.8 | 7.1 |  |  | 1.8 | -0.5 | 4.1 |  |
| 56-65 years | 7.5 | 5.2 | 9.8 |  |  | 3.8 | 1.4 | 6.2 |  |
| ≥66 years | 9.0 | 6.4 | 11.7 |  |  | 5.0 | 1.7 | 8.4 |  |
| **Highest education** |  |  |  | **<0.001** |  |  |  |  | **0.038** |
| Compulsory schooling/Vocational training | -2.7 | -4.5 | -0.9 |  |  | -1.7 | -3.4 | -0.02 |  |
| Upper secondary education | ref |  |  |  |  | ref |  |  |  |
| University education | -3.3 | -4.8 | -1.9 |  |  | -1.8 | -3.2 | -0.4 |  |
| **Language of questionnaire** |  |  |  | **<0.001** |  |  |  |  | **<0.001** |
| German | ref |  |  |  |  | ref |  |  |  |
| French/Italian | -5.0 | -6.7 | -3.2 |  |  | -3.3 | -5.0 | -1.5 |  |
| **Children <14 years in household** | |  |  | 0.974 |  |  |  |  |  |
| No | ref |  |  |  |  |  |  |  |  |
| Yes | -0.02 | -1.2 | 1.2 |  |  |  |  |  |  |
| **Employment status** |  |  |  | **<0.001** |  |  |  |  | 0.198 |
| Employed/In education | ref |  |  |  |  | ref |  |  |  |
| Other^b^ | 3.7 | 1.9 | 5.5 |  |  | 1.5 | -0.8 | 3.7 |  |
| **Job type** |  |  |  | **0.002** |  |  |  |  | 0.409 |
| Health Services | ref |  |  |  |  | ref |  |  |  |
| Essential services^c^ | -1.4 | -3.2 | 0.3 |  |  | -0.2 | -1.9 | 1.5 |  |
| Office jobs^d^ | -2.5 | -3.8 | -1.3 |  |  | -0.7 | -1.9 | 0.5 |  |
| Other^e^ | -1.7 | -3.9 | 0.4 |  |  | -1.8 | -4.4 | 0.7 |  |
| **Living situation** |  |  |  | **<0.001** |  |  |  |  | **<0.001** |
| Alone | -3.9 | -5.4 | -2.4 |  |  | -3.0 | -4.4 | -1.6 |  |
| Partner | ref |  |  |  |  | ref |  |  |  |
| Partner and children | -1.4 | -2.7 | -0.1 |  |  | -0.3 | -1.6 | 1.0 |  |
| Parents and/or children | -5.0 | -7.0 | -3.1 |  |  | -2.7 | -4.6 | -0.8 |  |
| Other situation^f^ | -5.4 | -7.4 | -3.4 |  |  | -3.6 | -5.5 | -1.7 |  |
| **Time since start of pandemic measures (days)** | -0.02 | -0.1 | 0.04 | 0.545 |  |  |  |  |  |
| **Physical distancing behaviour** |  |  |  | **0.008** |  |  |  |  | 0.412 |
| Physical distancing | ref |  |  |  |  | ref |  |  |  |
| (Self-)isolation | -2.3 | -3.8 | -0.8 |  |  | -0.9 | -2.4 | 0.5 |  |
| No physical distancing/Initial physical distancing | -1.1 | -2.4 | 0.3 |  |  | 0.0 | -1.2 | 1.3 |  |
| **Contact to person with COVID-19** |  |  |  | 0.098 |  |  |  |  |  |
| No | ref |  |  |  |  |  |  |  |  |
| Yes, assumed/confirmed | -1.2 | -2.6 | 0.2 |  |  |  |  |  |  |
| **Perceived COVID-19** |  |  |  | **<0.001** |  |  |  |  | **0.007** |
| No | ref |  |  |  |  | ref |  |  |  |
| Yes | -3.4 | -5.1 | -1.7 |  |  | -2.2 | -3.7 | -0.6 |  |
| **At risk for severe course of COVID-19** |  |  |  | 0.250 |  |  |  |  |  |
| No | ref |  |  |  |  |  |  |  |  |
| Yes | -0.9 | -2.3 | 0.6 |  |  |  |  |  |  |
|  |  |  |  |  |  |  |  |  |  |
| **Having person to ask for support** |  |  |  | **<0.001** |  |  |  |  | **<0.001** |
| No | -8.3 | -11.3 | -5.3 |  |  | -6.8 | -9.7 | -4.0 |  |
| Yes | ref |  |  |  |  | ref |  |  |  |
| No need for support | 5.3 | 4.2 | 6.3 |  |  | 3.9 | 2.9 | 4.9 |  |
| **Contact frequency with family and friends** |  |  |  | **<0.001** |  |  |  |  | **<0.001** |
| No, not enough contact | ref |  |  |  |  | ref |  |  |  |
| Yes, enough/No need for contact | 4.7 | 3.3 | 6.0 |  |  | 4.4 | 3.2 | 5.7 |  |
| **Frequency of information about COVID-19** |  |  |  | 0.130 |  |  |  |  |  |
| Daily | ref |  |  |  |  |  |  |  |  |
| Several times per week | -1.1 | -2.4 | 0.2 |  |  |  |  |  |  |
| Once per week or less | -1.4 | -3.2 | 0.5 |  |  |  |  |  |  |
| **Health literacy (score)** | 0.3 | 0.2 | 0.4 | **<0.001** |  | 0.2 | 0.1 | 0.3 | **<0.001** |

**Comments:** Higher scores of the MCS indicate better mental HRQOL. Multivariable linear regression models included sex, age at survey, and highest education level regardless of their significance in the univariable regression model, because we assumed *a priori* that these variables were important predictors of HRQOL. p values<0.05 are indicated in bold.

**Abbreviations:** CI, confidence interval; Coeff, coefficient; COVID-19, corona virus disease 2019; HRQOL, health-related quality of life; MCS, Mental Component Summary; ref, reference category.

**Footnotes:**

^a^ P values derived from *mi test* (in Stata) to perform joint tests that coefficients are equal to zero for a global effect of the categorial variable on mental HRQOL (MCS).

^b^ Other employment status includes persons who were retired, managing a household, seeking for a job, receiving disability insurance, or other forms of occupation.

^c^ Essential services include jobs within the areas of agriculture, manufacturing, waste management, construction; trade, transportation, gastronomy; education; social work.

^d^ Office jobs spans the fields of information and communication, finances, insurances, real estate; scientific and technical activities; administration; arts and other service activities.

^e^ Other jobe type includes persons who were retired, unemployed or not actively working.

^f^ Other living situation includes persons living in a shared apartment or other living arrangements.

**Figure S1:** Flow diagram for the CoWELL sample (n = 1581).


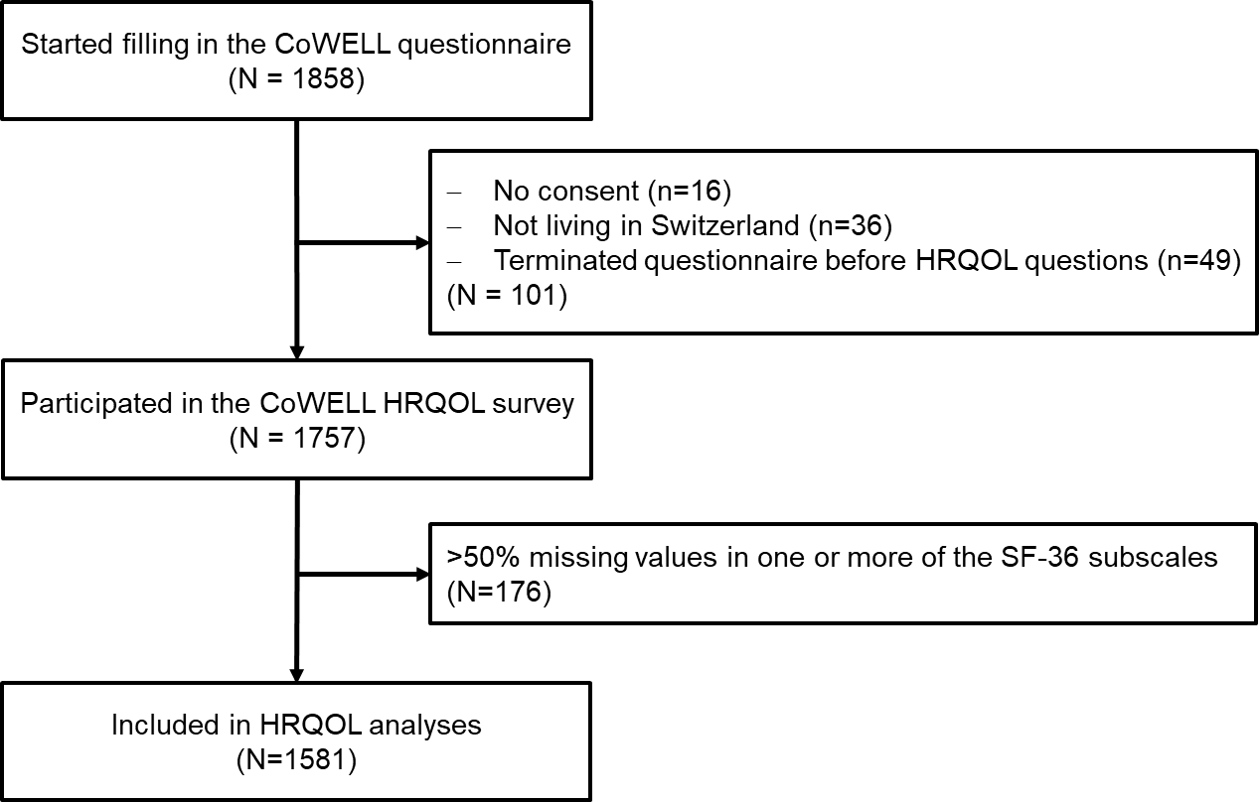

Supplement: Supplementary file 1 — Supplementary file1 (DOCX 125 KB) [file 11136_2023_3414_MOESM1_ESM.docx]
